# Seeking and encountering online information for menstrual health: a qualitative study among adolescent schoolgirls in Gianyar Regency and Denpasar City, Bali, Indonesia

**DOI:** 10.1080/26410397.2024.2445936

**Published:** 2025-01-08

**Authors:** Heather Suttor, Kadek Putri Yamayanti, Ni Luh Eka Purni Astiti, Tungga Dewi, Richard D. Chenhall, Ansariadi Ansariadi, Julie Hennegan

**Affiliations:** a PhD candidate, Melbourne School of Population and Global Health, University of Melbourne, 207 Bouverie Street, Victoria, 3010, Melbourne, Australia; Research Assistant, Maternal, Child and Adolescent Health Program, Burnet Institute, Melbourne, Australia.; b Research Assistant, Perfect Fit Indonesia, PT Putri Fajar Inspirasi, Bali, Indonesia; c Executive Director, Perkumpulan Keluarga Berencana Indonesia (PKBI) Daerah Bali, Bali, Indonesia; d Co-founder, Executive Director, Perfect Fit Indonesia, PT Putri Fajar Inspirasi, Bali, Indonesia; e Professor, Melbourne School of Population and Global Health, University of Melbourne, Melbourne, Australia; f Director, Centre for Epidemiology and Population Health Studies, Hasanuddin University, Makassar, Indonesia; g Senior Research Fellow, Maternal, Child and Adolescent Health Program, Burnet Institute, Melbourne, Australia; Honorary Research Fellow, Melbourne School of Population and Global Health, University of Melbourne, Melbourne, Australia

**Keywords:** menstrual health, adolescent health, online health information, social media, qualitative, Indonesia

## Abstract

Sufficient and accurate information is a requirement for menstrual health and supports adolescents in realising their human rights. As mobile connectivity increases globally, many young people may seek or encounter menstrual health information online through web-based platforms, social media, or health apps. Despite the relevance of online information, menstrual health research and programming have focused on formal and school-based learning. Using a participatory and ethnographic approach over seven months from November 2022 to June 2023, this qualitative study explores how adolescent girls between 13 and 15 years of age in junior high school in two districts of Bali, Indonesia, access and use online information for menstrual health learning. Findings are from 20 group discussions; sessions were held five times with each group across four schools. Fourteen participants also completed solicited diaries, and five participated in interviews. Data are also drawn from participant observation in schools and community spaces. We found that informal online information is a significant source of menstrual health learning and is accessed through active searching and incidental encounters. The motivations to access and use online information were specific to participants’ menstrual health needs. We found that online information presented opportunities for personalised and convenient learning. However, it also presented risks associated with excessive and inappropriate information that caused worry and reinforced menstrual myths. Our findings highlight the need to account for informal online information in future research and programming on menstrual health, particularly in contexts with a high level of mobile connectivity among young people.

## Plain language summary

Good quality information helps adolescents with their menstrual health. It supports them in realising their human rights. Many adolescents find information on menstrual health online through websites, social media, or health apps. Online information is increasingly important. But we know little about how adolescents access and use it for menstrual health. We conducted a qualitative study in junior high schools in two districts of Bali, Indonesia. 24 school girls aged 13–15 took part. We wanted to understand how they accessed online information and what they learnt from this about menstruation. We carried out five group discussions in each of four junior high schools. We also did five in-depth interviews, a solicited diary activity and participant observation. Our study confirmed that online sources were important for menstrual health information. Participants shared that they looked for menstrual health information through search engines. They also came across it via social media, especially the video platform TikTok. Many participants saw online information as positive. They felt it offered personalised, private, entertaining and convenient information. But there were also risks. Excessive information caused them to worry and misinformation reinforced menstrual myths. Our findings highlight the need for menstrual health programming and policy to take into account the role of online information. It would also commit to understanding the lived experiences of adolescents. Critical health and digital literacy could be part of puberty and health education. This would be very helpful in countries like Indonesia with growing digital engagement.

## Pernyataan Bahasa Sederhana

Informasi berkualitas memberikan remaja akses terhadap kesehatan menstruasi sekaligus mendukung kesadaran mereka akan hak asasi manusia. Mayoritas remaja mengakses informasi kesehatan menstruasi secara daring melalui situs web, media sosial, atau aplikasi kesehatan. Meskipun informasi daring semakin relevan, pemahaman kita tentang bagaimana remaja mengakses dan menggunakan informasi daring untuk kesehatan menstruasi sangat jauh dari kata cukup. Penelitian ini dilakukan dengan kajian kualitatif pada 24 remaja putri berusia 13-15 tahun di sekolah menengah pertama di dua kabupaten di Bali, Indonesia (Kabupaten Gianyar dan Kota Denpasar). Penelitian ini bertujuan untuk memahami bagaimana remaja mengakses informasi daring dan apa yang mereka pahami dari informasi tersebut terkait menstruasi. Sebanyak lima kegiatan diskusi kelompok dilakukan di setiap empat sekolah menengah pertama. Selanjutnya, dilakukan lima wawancara mendalam, aktivitas buku harian singkat, dan observasi partisipan. Hasil dari penelitian ini menegaskan bahwa sumber daring berperan penting untuk informasi kesehatan menstruasi. Partisipan menyatakan bahwa mereka secara aktif mencari informasi kesehatan menstruasi melalui mesin pencari (Google) dan/atau menemukannya secara tidak sengaja melalui media sosial, khususnya platform video pendek TikTok. Sebagian besar partisipan berpendapat bahwa informasi daring membawa dampak positif. Selain itu, mereka menyatakan bahwa platform daring menawarkan informasi yang bersifat pribadi, rahasia, menghibur, dan praktis. Namun, terdapat risiko dari informasi daring tersebut. Terkadang terdapat informasi yang berlebihan yang membuat mereka cemas atau informasi yang salah yang memperkuat mitos tentang menstruasi. Dari penelitian ini dapat disimpulkan bahwa pentingnya program dan kebijakan kesehatan menstruasi untuk lebih memperhitungkan peran informasi daring dan berkomitmen untuk memahami pengalaman hidup remaja. Program atau kebijakan lebih lanjut dapat mengintegrasikan literasi kritis tentang kesehatan dan digital ke dalam pendidikan kesehatan dan pubertas, khususnya di negara-negara yang mengalami perkembangan digital yang pesat, seperti Indonesia.

## Résumé

Des informations suffisantes et exactes sont indispensables pour la santé menstruelle et aident les adolescentes à réaliser leurs droits humains. À mesure que la connectivité mobile augmente dans le monde, de nombreux jeunes peuvent rechercher ou trouver des informations sur la santé menstruelle en ligne via des plateformes internet, des réseaux sociaux ou des applications de santé. Malgré la pertinence des informations en ligne, la recherche et les programmes en matière de santé menstruelle se sont concentrés sur l’apprentissage formel et scolaire. Utilisant une approche participative et ethnographique sur sept mois, de novembre 2022 à juin 2023, cette étude qualitative examine comment les adolescentes âgées de 13 à 15 ans scolarisées dans des établissements secondaires du premier cycle dans deux districts de Bali, en Indonésie, obtiennent et utilisent des informations en ligne pour en savoir plus sur la santé menstruelle. Les résultats proviennent de 20 discussions de groupe; des séances ont eu lieu cinq fois avec chaque groupe dans quatre écoles. Quatorze participantes ont aussi complété des journaux personnels (*solicited diaries*) et cinq ont participé à des entretiens. Les données sont également tirées de l’observation des participantes dans les écoles et les espaces communautaires. Nous avons constaté que les informations informelles en ligne constituent une source importante d’apprentissage sur la santé menstruelle et qu’elles sont accessibles par des recherches actives et des rencontres fortuites. Les motivations pour avoir accès aux informations en ligne et pour les utiliser étaient spécifiques aux besoins des participantes en matière de santé menstruelle. Nous avons remarqué que l’information en ligne présentait des occasions d’apprentissage personnalisé et pratique. Néanmoins, elle comportait aussi des risques associés à une information excessive et inadaptée qui suscite des inquiétudes et renforce les mythes autour des menstruations. Nos résultats mettent en évidence la nécessité de tenir compte des informations informelles en ligne dans les recherches et programmes futurs sur la santé menstruelle, en particulier dans les contextes présentant un niveau élevé de connectivité mobile chez les jeunes.

## Resumen

La información suficiente y veraz es un requisito para la salud menstrual y apoya a las adolescentes en ejercer sus derechos humanos. A medida que la conectividad móvil aumenta a nivel mundial, muchas personas jóvenes posiblemente busquen o encuentren información digital sobre salud menstrual por medio de plataformas en internet, redes sociales o aplicaciones sobre salud. A pesar de la relevancia de la información digital, las investigaciones y los programas de salud menstrual se han centrado en el aprendizaje formal y escolar. Utilizando un enfoque participativo y etnográfico durante siete meses, desde noviembre de 2022 hasta junio de 2023, este estudio cualitativo explora cómo las adolescentes entre 13 y 15 años, en escuelas secundarias en dos distritos de Bali, Indonesia, obtienen y utilizan información digital para aprender sobre salud menstrual. Los hallazgos provienen de 20 discusiones en grupos; se llevaron a cabo sesiones cinco veces con cada grupo en cuatro escuelas. Catorce participantes también llevaron diarios solicitados y cinco participaron en entrevistas. Además, se obtuvieron datos por medio de la observación participante en escuelas y espacios comunitarios. Encontramos que la información informal digital es una fuente significativa del aprendizaje sobre salud menstrual y es obtenida por medio de búsquedas activas y encuentros incidentales. Las motivaciones para obtener y utilizar información digital estaban relacionadas específicamente con las necesidades de salud menstrual de las participantes. Encontramos que la información digital presentaba oportunidades para el aprendizaje personalizado y conveniente. Sin embargo, también presentaba riesgos asociados con información excesiva e inadecuada que causaba preocupación y reforzaba los mitos menstruales. Nuestros hallazgos destacan la necesidad de considerar la información digital informal en futuras investigaciones y programas sobre salud menstrual, en particular en contextos con alto nivel de conectividad móvil entre jóvenes.

## Introduction

Many young people seek and encounter health information online through web-based platforms and social media.^1–4^ Often, this learning has informal qualities and is “self-directed”, occurring outside or parallel to school-affiliated health education programmes.^5^ Access to sufficient and accurate information is a right and a fundamental requirement for achieving menstrual health, the “complete physical, mental and social well-being” during the menstrual cycle.^6^ However, despite the growing importance of online information for menstrual health among adolescent populations, research and programming have prioritised formal and school-based learning, with minimal attention to the increasing role of online information.

### The current state of reported menstrual health learning and education

Interventions to improve the provision of menstrual health information have focused on delivering education through updated national curriculums or extra-curricular education activities facilitated by external organisations. ^7^ In Indonesia, whilst menstruation education is provided in primary and junior high school through the National School Health Programme (UKS), delivery and content quality vary greatly, and menstrual health may be excluded entirely from health education. ^8,9^ When menstrual health content is taught, it is often theoretical, biological, and a small component of puberty education. Teachers may feel the topic is taboo, be underprepared or lack the resources and time to share information on menstruation beyond the basic functions and anatomy of the reproductive system.^9^ Extra-curricular activities offered through external organisations and the development of localised digital health tools, such as the Oky App (UNICEF), aim to address these challenges and provide out-of-school opportunities for young people to engage with menstrual health learning. However, many young people in Indonesia continue to have limited opportunities to develop and apply comprehensive menstrual health knowledge. Accordingly, certain taboos and menstrual myths persist, for example, restrictions on personal hygiene such as bathing and hairwashing, visiting certain places such as rice fields or disposing of used pads without thorough washing.^9^ This is mirrored globally; qualitative research and small-scale cross-sectional surveys have repeatedly found that many adolescent girls lack relevant information about menarche and menstruation, potentially increasing anxiety and shame around menstruation or promoting unhealthy or stressful menstrual management practices.^10–12^

Alongside formal education, family members play a crucial role in young people’s menstrual health learning. In Indonesia, mothers are frequently identified as a central source of menstrual information.^9^ Information from family members and learning in informal spaces, such as at home or in the community, can be variable. While family members are often preferred sources of information, culturally situated connection and care,^13,14^ qualitative studies have equally highlighted that this knowledge sharing can be absent, vague, and maintain harmful norms or restrictions surrounding menstruation.^10,15^

### Online health information

Scholars and practitioners have explored the risks and benefits of online health information. There has been a focus on misinformation, exploring challenges and offering possible recommendations,^16^ and emphasising the need to safeguard young people. ^17^ Numerous qualitative and quantitative studies demonstrate the prevalence of health mis/disinformation through social media on various topics. ^18,19^ While the prevalence of misinformation related to menstrual health has not been widely assessed, a descriptive evaluation study found that online information on menstrual pain (dysmenorrhoea) had mixed credibility and quality.^20^

Beyond possible risks, there is growing acknowledgment that online spaces, especially social media, also have the potential to act as catalysts for learning and can promote dialogue, activism, and communities of care.^21–23^ Furthermore, recent research has demonstrated the necessity of direct engagement with young participants to understand the situated and multivalent role of digital technologies in their lived health and well-being learning experiences.^4,24–26^

### Digital engagement in Indonesia

While gaps persist, mobile internet coverage and connection are high across many regions of Indonesia, opening up online information as a source of menstrual health information for millions of young people.^27^ Many young Indonesians are frequent mobile internet users and were nudged into a predominantly digital learning environment during the COVID-19 pandemic due to extensive school closures.^28,29^ A 2020 survey study among students in urban and rural Java found that from the age of 13, more than 90% of respondents owned a smartphone, regardless of location or socioeconomic status, with regular use of social media apps.^30^ Indonesia is reported to have the second-highest number of TikTok users globally, with 126 million people, or just under half the total population using the platform, many of whom are young people.^31^ High levels of social media uptake have prompted research investigating the effectiveness of sharing relevant health education using this medium.^32^ Specific to young populations, a recent pre- and post-test experimental study among junior high school students in Semau Island, East Nusa Tenggara (NTT), a small island district in a province in eastern Indonesia, found TikTok media improved adolescents’ sexual and reproductive health knowledge and attitude scores compared to paper-based leaflets.^33^

### The present study

To the best of our knowledge, there is limited research on the role of online and digital learning for menstrual health, specifically research that considers informal learning and young people's perspectives and lived experiences. Further evidence is required to understand if current research that explores young people’s use of online information in other or more general health domains^1,4,25,34,35^ can be applied to menstruation, particularly given the widespread stigma associated with menstruation in many contexts in the Asia Pacific region, including in Indonesia.^7^

The research presented here is one component of a qualitative doctoral research project investigating the connections between digital tools and young people’s menstrual health experiences in Bali, Indonesia. For the study presented in this paper, we aim to understand how young people in Bali, Indonesia, access and use online information for menstrual health. Specifically, we responded to the following questions: (1) What is the role of online information in menstrual health learning among adolescent girl participants in Bali, Indonesia? (2) How do participants access information on menstrual health online? (3) How do participants integrate online menstrual health information into their menstrual practices, and how does this inform their menstrual experiences?

## Methods

### Design

We took a participatory and ethnographic approach using qualitative data collection techniques of group discussions, observations, interviews, and diaries with participants in two districts, Denpasar City and Gianyar Regency, over seven months from November 1, 2022, to June 7, 2023. The research was designed collaboratively with the research team in Indonesia (KPY, AA, HS) and Australia (JH, RC) and in-country partners EP of Kisara Perkumpulan Keluarga Berencana Indonesia Bali (PKBI Bali), the youth-focused Balinese chapter of Indonesian planned parenthood association (IPPA), and TD of Perfect Fit Indonesia, a social enterprise that produces sustainable period products and delivers menstrual health education. In-country partners contributed their expertise on sexual and reproductive education in the Balinese and broader Indonesian contexts. Data were collected by HS, an Australian woman, a PhD researcher with a background in anthropology and global health research and implementation, and the co-researcher KPY, a young Balinese woman and translation studies researcher.

### Setting

Data collection was conducted in Bali, Indonesia, where both partner organisations were based and conducted activities. Bali is an island province in central Indonesia, home to 4.3 million of Indonesia’s 275 million population.^36^ Bali is a majority Hindu province; according to Census data from 2010, 83% of the population identified as Balinese Hindu and 13% identified as Muslim, with small populations of Christians (2%), Buddhists (0.5%) and Confucianists (0.01%).^37^ Data collection focused on two districts in Bali: Denpasar City and Gianyar Regency. Denpasar City is a densely populated urban district and the capital of Bali, while Gianyar Regency includes urban, rural and peri-urban settings.

### Sampling & recruitment

Data were collected in junior high schools or Sekolah Menengah Pertama (SMP), covering grades 7–9 with an average age range of 13–15 years. Together with the in-country partners, we identified junior high schools with planned or ongoing menstrual health activities throughout the research period. After this initial selection, we focused on schools within a 1.5-hour drive of each other and the local partner’s office due to travel and resource limitations. From this, we selected four schools to encourage a representative sample. This included three government schools and one private school. Three schools were in urban areas, and one was in a rural area. After selection and initial contact, KPY and HS met with school leadership and teachers to introduce the research process and objectives and to seek approval to conduct the research. All four initially selected schools agreed to participate in the research.

Sampling and recruitment of participants were conducted in close partnership with Kisara/PKBI Bali and the school staff. Teachers supported recruitment by selecting students from grades 7–9 to attend an introductory information session based on their identification as an adolescent girl or *“remaja putri”* and their availability at extracurricular time. No other inclusion criteria were specified. The limited criteria were intended to encourage a diversity of menstrual and digital experiences and to reduce exclusion or the need for potential participants to state their menstrual status.

### Data collection

Data collection started after an initial scoping and familiarisation period and identification and recruitment of schools and participants (August-November 2022). The seven-month duration of the study (November 2022–June 2023) allowed us to understand participants’ experiences over a suitable time, ensuring sufficient breaks between sessions and reducing overburdening participants and schools. The duration loosely followed the Indonesian school year, with all activities starting alongside the local partner’s implementation of menstrual health activities and concluding after final examinations in June 2023. Group discussions and interviews with participants took place in the four junior high school sites. Observations were conducted in various community spaces, including schools where partner organisations conducted education activities.

#### Group discussions

Data collection focused on a series of five group discussions at each school site, with 20 group discussions in total. The participants remained consistent over the research period, and discussion themes and activities were built upon one another. The research team planned for up to six group discussions, allowing for flexibility. After the initial three sessions, we understood that five sessions were optimal for establishing a trusting and productive discussion environment.

Group discussions usually lasted 1.5 hours and centred around a theme and 1–2 activities, using techniques such as mapping, ordering, walkthroughs, vignettes, and role-play. Sessions were conducted in Bahasa Indonesian with joint facilitation from KPY and HS. Session topics were initially developed through a literature review and consultations with partners and evolved iteratively in response to participants’ priorities and ongoing data analysis. We planned for the initial discussion group to cover the sources and content of menstrual information. This was selected as the first, as it did not require participants to discuss their menstrual experiences or status in depth. It also helped us to establish a baseline understanding of participants’ questions and concerns and the potential role of digital tools. Subsequent sessions explored menstrual experiences, menstrual tracking and the content and role of social media. Whilst activities were consistent across the groups, content and delivery were tailored to each group based on findings and group dynamics.

All group discussions were audio recorded with participants’ permission. Photo data and written data were also collected from activity sessions. Sessions are labelled throughout this report as “GD” with session number (1–5) and the site code (A – D).

#### Diaries

All participants enrolled in the study and participating in group discussions were invited to complete the diary activity. Fourteen participants completed written diaries over three months outside of school hours. Participants were given a bag containing a paper diary, pens and sticky notes. Participants were encouraged to write freely about their menstrual and online experiences. To support participants, prompts were sent via WhatsApp every 2–3 weeks, depending on holidays and exams. Prompts offered ideas and guidance, such as, “You can write about something you learned about your body or menstruation. How did you learn about it? How did it make you feel?” Participants brought diaries to sessions where they could select which entries they would like to be photographed for transcription. This approach helped to emphasise participants’ ownership of their data and that they were invited to participate in the diary activity regardless of whether they wished to share their entries.

#### Interviews

All participants enrolled in the study and who participated in group discussions were invited to an individual in-depth interview. Five participants volunteered to take part in individual in-depth interviews. We conducted interviews at school, at the end of the data collection period, and after final exams. Interviews lasted approximately one hour and were conducted by HS and KPY, with KPY translating. All interviews were audio recorded with participants’ permission.

#### Observation

HS conducted observations during group discussions, focusing on group dynamics, body language, and engagement with digital tools (solicited and unsolicited). We also conducted observations during our partner organisations’ education activities. HS continued informal participant observation in her daily activities. Observations were recorded in field note journals.

### Data analysis

Analysis was inductive and iterative, starting early in the data collection process and continuing after the final activities.^38^ HS conducted analysis, with feedback from the research team and partners throughout. Data analysis started with writing post-activity field notes and memos. This supported the early identification of themes and helped refine the research questions, data collection techniques, and activities. Repeating activities and discussing observations with participants and partners supported the analysis of initial findings.

Data from group discussions and interviews were transcribed by KPY from recordings directly into English. The written data from sessions and diaries were transcribed by HS in Bahasa Indonesia, and the translation was done by KPY. Once available, translated and de-identified data were uploaded to NVivo for management and initial open coding. Toward the end of data collection, HS continued to develop and consolidate existing codes and conduct further in-depth coding to develop themes.

### Ethics

This project was granted ethics approval from the University of Melbourne (ref: 2022-23745-27644-3) (09/05/2022) and from the Ministry of Research, Technology and Higher Education, Indonesia (BRIN) (ref: 193 /KE.01/SK/8/2022) (29/08/2022). Following Australian guidance as detailed in the National Statement of Ethical Conduct in Human Research,^39^ informed consent was sought from all adolescent participants and parents. Interested students were invited to a voluntary information session on the study, where they were provided with a plain language statement, as well as an information document and consent form for parents to sign. Adolescent participants were asked for written consent during the first session. The research team emphasised a continuous approach to consent throughout the research process.

Participants were provided with meals and snacks. They also received two gifts in appreciation for their time: a set of four reusable pads, a wash bag (GD 3), and a tote bag with a diary and stationery (GD 2), which they could use for the diary activity if desired. Participants chose their pseudonyms for diaries and interviews.

## Results

### Participant characteristics

A total of 24 participants volunteered for the study across four schools (see Table 1), with 20 completing at least three group discussions. On average, five participants attended each group session. One participant withdrew from the study, reporting changes in availability and parental permission. While drop-out was minimal, some participants were unavailable for all five sessions. Five participated in interviews, and 14 completed diaries.

At the time of the research, all participants reported starting menstruation, having a personal smartphone with a mobile internet connection, and using their phones daily. We observed and discussed varied access to internet data and WIFI among the participants.

### Menstrual health information sources

**Figure 1.**
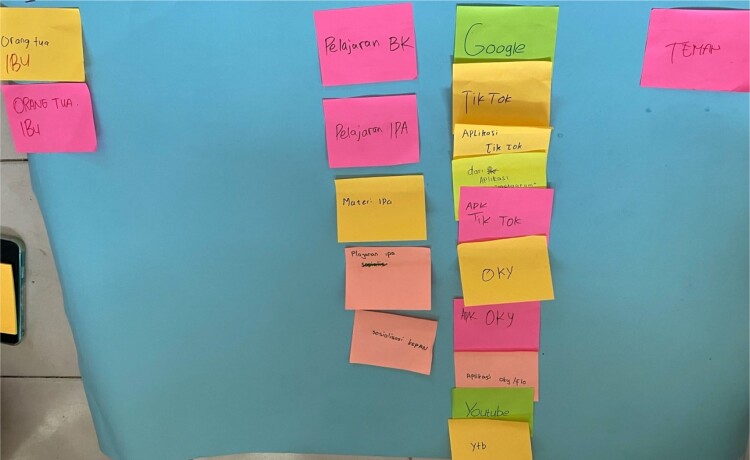
Example of mapping GD 1 from Site B

Participants mapped sources of menstrual health information, as shown in Table 2 and the example in Figure 1. Across the four groups, the sources of information were similar, emphasising the role of families, particularly mothers, and internet and digital sources. However, the types of information delivered or sought differed across the sources and varied between participants depending on their needs and social and material environments.

**Table 1.** Participant characteristics

Site	Total	Site A	Site B	Site C	Site D
Number of participants	(*n* = 24)	6	7	5	6
Grade level		8–9	9	7–8	7
Age		13–15	14–15	12–14	12–13
**Participants per activity**					
Group discussion	24	6	7	5	6
Individual in-depth interviews	5		4	1	
Diary completed	14	2	5	3	4

**Table 2.** Details the sources, sites and types of information participants mapped during GD 1

Source	Site/tools	Types of information
**Family**	Mothers, sisters, female cousins, or aunts (female family members)	Care, practical information, food and drink restrictions, personal hygiene restrictions
**Internet and digital**	Google, TikTok, Instagram, Twitter YouTube, Wiki How, Menstrual tracking or education apps (Oky App, Flo App)	Practical and “medical” information and remedies, symptom searching, entertainment, verification of myths and restrictions
**School**	Natural sciences (“IPA”), counselling (“BK”), Religion, extracurricular activities non-government organisations like PKBI Bali, and Youth Red Cross, school science books.	Information on puberty and reproduction, practical information on hygiene and diet, religious rules and restrictions, introduction of apps
**Friends**	Friends (boys and girls)	Sharing and support, reminding of period dates, sharing myths, teasing

#### Informal online and digital information

For many participants, online and digital sources were seen as accessible and trustworthy, offering a complement or an alternative to information from parents, friends, or school. Online learning was self-directed, and participants expressed a desire, driven by both interest and need, to learn more about their menstrual health.

Online learning for menstrual health occurred primarily through smartphones. In most discussion groups and during observations in other schools, participants and students had their phones “on hand” during school hours. Only one school site restricted the use of smartphones at school. During interviews, participants discussed their screen time, some pushing their phones towards us to see the breakdown of different uses, others noting a screen time of 7-8 hours – or jokingly saying *“24 hours”* to imply a continuous use and presence (Interview Tasya, grade 9). Within the groups, participants discussed how they only accessed the internet through their smartphones, including for school-related communication, with only one participant explicitly mentioning using a laptop for study purposes.

### Pathways to online MH learning: active information-seeking and incidental encounters

*“This morning I checked my phone looking for information about menstruation on Google. Sometimes Googling and I ask my mum. In the afternoon, I watched TikTok then it popped up about menstruation and I read it.” (Diary, Zahra, Grade 8)*

Zahra’s diary excerpt reflects the two online information-seeking pathways participants discussed in the groups. Simply, she used her phone to actively seek information about menstrual health, and she also encountered menstrual health information incidentally through social media. While these pathways inform and interact with one another, participants tended to separate actions and practices related to Google from those related to social media applications, discussing the motivations and activities associated with each.

*Active information seeking through Google*

#### - Do they use it?

Participants highlighted the role of active online information-seeking for menstrual health learning. We define active or intentional information as information that is sought, usually to meet a specific need or respond to a question. For participants, this action entailed intentional and directed searches, mainly through Google, and then tracing the relevant information through websites. On occasions, this active searching occurred through YouTube (video platform) or TikTok, through the search function.

The frequency of searching through Google for menstrual health information varied. Some participants reported occasionally searching for information *“just when we need it”* (GD 1, site C) or *“sometimes”* (GD 5, site A), and others said they would seek out information on health or their bodies every day, both at school and at home. Some participants reported downloading specific menstrual health apps, such as the Flo App or Oky App, menstrual cycle tracking tools that also provide menstrual health information. However, these were used less frequently than the regular use of Google or social media throughout the menstrual cycle.

Google could be on hand when needed, with one participant stating, “*My HP [mobile phone] is always with me, so Google is the answer”* (GD 2, site D).

#### - How do they search?

Participants employed targeted search strategies to find suitable information. When searching, they preferred complete sentences rather than keywords, as they believed this would result in more detailed and precise answers. As one participant shares, she will type a complete sentence like: “*How to take care the reproductive organs?”* Otherwise, she explains, “*the result will not be clear”* (GD 4, site D).

Participants rapidly evaluated Google search results and then selected the information source they understood as correct and aligned to their needs. During a walk-through activity (GD 5), participants showed us how they selected the first answer, as it was often seen as the most relevant. If the initial results were not deemed as suitable, they would scroll through to find the most appropriate solution; as Julie described, her process involved *“scrolling everything but I chose the one that I think was closest to my case”* (Interview, grade 7). Or, as another participant suggested, *“I just focus on the thing that I think is correct”* (GD 5, site A). The *People Also Ask* section, a Google-generated summary of questions and answers, was sometimes seen as sufficient.

Participants frequently selected recognisable and commercial health tech platforms, such as Halodoc, private Indonesian hospitals, or instructional websites, such as WikiHow. Partners from PKBI Bali shared that there were limited comprehensive and suitable web-based information resources for menstrual health in Bahasa Indonesian. This gap may result in a reliance on commercial provider websites. Shorter articles, clear subheadings, and informative “how to” diagrams made the information more digestible, which is characteristic of a preferred website, WikiHow. The content of some sites was seen with occasional scepticism, demonstrating participants’ awareness of online misinformation. For example, participants noted cases when results from Google or sites such as Halodoc were less trustworthy and contained *“hoaxes”* - so *“it is better to consult the doctor directly”* (GD 5, site B).

#### - What do they search for?

Questions from discussions and a Google Question generating activity (GD 5) sat across four domains, as shown in Table 3. These questions reveal participants’ knowledge gaps. Equally, they demonstrate an interest in general and personalised information on the menstrual cycle and menstrual experiences, practical information to inform their personal needs and practices, and information to confirm or debunk menstrual restrictions and rules.

#### Incidentally encountering information through social media (TikTok)

*“In TikTok, I saw on my FYP [for you page] about menstruation and the cycle. I didn’t look for it, it just appeared.”* (GD 1, site C)

**Table 3.** Examples of questions reported from a Google brainstorm activity (GD 5)

Primary category	Question examples
How and why (general)	*How long is the normal menstrual period? At what age does menstruation start in teenage girls? Is menstruation very important for women?*
How and why (personal)	*Why do I have a stomach ache [cramps] when I am menstruating? Why at the end, does blood suddenly comes out again? Is it normal if you don't have your period for a month?*
Practical	*What is a sleeping position, so it [blood] doesn't leak? How to get rid of stomach pain [menstrual cramps]? How to control mood swings during menstruation?*
Verifying information or restrictions	*Is it okay to eat spicy food during menstruation? Is it okay to sleep a lot during menstruation? Does drinking cold water make your blood clot?*

##### - Do they use it?

The short-form video platform TikTok created numerous encounters with menstrual health information, providing opportunities for incidental learning. We understand incidental learning as occurring during activities where learning is not the primary objective, for example, during play, self-care, or entertainment. Interview participants told us how they used TikTok daily, sometimes for several hours. Whilst encounters with menstrual health information were usually not the primary motivation for using social media, they were neutral or desirable encounters. This was demonstrated through participants’ engagement – viewing and occasionally liking – the content, as well as their recall of the content during group discussions.

Whilst TikTok was the most common platform among participants, not all were regular users, and many simultaneously used other platforms, particularly Instagram. A few preferred YouTube or Twitter for accessing menstrual health information. There was also a discussion around the downsides of social media like TikTok, demonstrating the diversity of online experiences and participants’ emerging critical engagement with the platform.

##### - How did they encounter it?

Participants generally encountered menstrual health content on TikTok through their “for you page” or FYP, the platform’s central and personalised landing page.^40^ As one participant shared, “* … TikTok. I just scrolled and there was an FYP about menstruation”* (GD 2, site D). Users’ content is shaped by the app’s proprietary algorithm rather than their social network.^41^ Online activities and interactions with content through actions such as watching, liking, sharing, or commenting can, as well as language and location, further personalise the user's experience and may increase the likelihood of users encountering menstrual health content.^42^ One participant demonstrated this flow, “*I just liked the video but then, another video popped up in TikTok. So I just watched it”* (GD 4, site C).

Whilst TikTok was repeatedly referenced as a primary information source, many participants noted that menstruation-related content was infrequent. However, when menstrual health videos did “pop up”, they would watch and recall the content but rarely follow the accounts or save the videos.

##### - What did they encounter?

The menstrual health-related content participants discussed was an assortment of educational videos on the reproductive system, as well as ghost stories about blood disposal. However, the TikTok content participants shared during a “show and tell” activity (GD 5) was predominantly educational and informative. Content included videos of fake blood moving through models of the reproductive organs to creators theatrically debunking common menstrual myths or “*mitos dan fakta*”. The content was delivered in Bahasa Indonesian and was relevant to participants’ menstrual practices; for example, the videos depicted disposable single-use pads over other menstrual materials or referenced common myths or restrictions in Indonesia. While creators were mostly female, there was occasional content from male creators, often resulting in laughter from the groups. As a group, we assumed that most creators were Javanese or based in Java, this was supported by their handles and hashtags. We observed that some creators appeared as nurses or doctors by wearing stethoscopes, scrubs, or lab coats. Some creators provided references to their qualifications and citations for the information they presented. However, for others, the guesswork of credibility was left up to the user.

### How online information informed the menstrual experiences of participants

Participants used information from both active information-seeking and incidental encounters to inform their menstrual experiences and practices. We developed three themes reflecting how information was used: debunking rules and restrictions and reinforcing taboos, making sense of, or complicating menstrual experiences, and learning and testing new practices.

#### Debunking “everyday” menstrual restrictions or reinforcing myths and taboos

Participants described how the information they sought (Google) and encountered (TikTok) shifted the ways they imagined and responded to certain restrictions and myths associated with menstruation: debunking *and* reinforcing.

Participants shared how they would draw on online information to contest existing information on restrictions. Restrictions were generally related to diet or personal hygiene and shared through parents, siblings, and peers. The consequences of these restrictions were usually physiological and individual. We noted that online information helped participants feel more comfortable to question restrictions, especially with parents, as it provided evidence to support their challenges. As one participant shared:

**Participant:** *so, my father told me that I cannot wash my hair when menstruating.*

**KPY:** *oh, okay, then?*

**Participant:** *then I told my father that Google said it was okay.*

(GD 5, site C)

Or as another participant told us: *“[mum says] don’t drink ice when you’re menstruating. Then I said this to mum, ‘that is a hoax mum’. I then searched for it in Google and drank it”* (GD 3, site C).

Google supported participants to triangulate and fact-check the assumed myth. This was often followed by testing the information in their bodies before accepting it and integrating it into their daily practices. For example, one participant explained, “*For the water, I believe TikTok since I tried to drink cold water and nothing happened”* (GD 2, site B).

However, sometimes the process of testing reinforced rather than debunked the restriction. We discussed this in relation to avoiding hair washing during menstruation – a common menstrual myth across Indonesia. For example, a participant told us that after triangulating multiple sources (mother, friend, Google), her most influential learning was experiential:

*“I asked my mum whether it was ok or not to wash my hair when menstruating, Mum said it is okay, but I also asked my friend and she said she could not. I asked why not. My friend had no answer, then I searched it on Google. Google said that it is okay to wash your hair. But then, I tried it when menstruating. I washed my hair and my menstruation was so quick.”*

(GD 5, site C)

Participants discussed trusting online information more than family members when it came to debunking everyday restrictions. As one participant shared:

**Participant:** *is it okay to drink cold water when menstruating? Google said yes but mum said no. After my mum said so, I directly searched it on Google, and found that was a myth.*

**KPY:** *which one do you believe?*

**Participant:** *Google.*

(GD 5, site C)

Trust was frequently linked to the idea that Google was “worldwide” (GD 1, site C) and contained an expanse of information from figures such as doctors or medical institutions.

Alongside the “everyday” restrictions related to food, drink, and personal hygiene, online information, especially through social media, contributed to the reinforcement of *pemali*, a shared Bahasa Indonesian term that can be translated to taboo. *Pemali,* or taboos, are traditional or customary social proscriptions where the consequences of violations can be both individual and collective and are often sanctioned by a supernatural influence.^43,44^ One recurring example was related to the risk of ghost encounters if participants did not wash menstrual materials to remove blood and dispose of materials in an appropriate place.

**Participant (a):** *yes. Like throwing the pads not in the appropriate place.*

**Participant (b):** *then the kuntilanak [female ghost] will eat the blood*.

**Participant (a):** *and follow you. Haha*

**HS:** *in TikTok?*

**Participant (b):** *yeah*

**HS:** *pop up or searching?*

**Participants (all):** *pop up!*

(GD 5, site D)

While the content participants discussed was perceived as entertaining, using storytelling and engaging audio and visuals, it also appeared to reinforce taboos related to menstruation.

Specifically, these encounters encouraged continued compliance and sometimes invoked worry or fear among participants. Numerous participants shared that they preferred to avoid the risk of encountering the ghost by continuing to wash their pads to remove traces of blood and by ensuring “appropriate” disposal. They suggested that some content encouraged them to be more “diligent” (GD 2 – Site A) in their cleaning or supported the relevance of their existing practice. However, the washing of pads was also mentioned in relation to privacy (removing visible traces of blood) and hygiene (removing the risk of odours) by some participants, indicating different practices and intersecting motivations between participants and age groups.

We also note that in Bali, there are numerous contextually specific religious restrictions during menstruation, such as entering a temple or participating in ceremonies while menstruating. Such restrictions were notably absent from any online information. This could be partially attributed to the lack of Balinese creators; however, it is also likely that any negotiation of religious restrictions is delicate and often personal or familial.

#### Informing and complicating menstrual experiences

Participants shared how informal online information helped to make sense of their menstrual symptoms and experiences, like pain, blood consistency and flow, or mood swings. For example, one participant told the group, “*In TikTok, there are so many doctors who make videos about menstruation, like what will happen during the menstrual cycle, like white discharge”* (GD 4, site C)**.** Information was accessed by “scrolling” TikTok looking for “*something related to blood like how to differentiate between red and brown blood”* (GD 3, site B) or actively searching through Google, as Alexa explained; “*On the 2nd day, my stomach suddenly hurt very badly. Then I searched on Google what [was] the cause of the pain”* (Diary, grade 7). There was often an emphasis on wanting to understand what was a “normal” symptom or experience, as Juanka expressed in her diary:

*“So, if I am menstruating sometimes, I feel pain in my stomach and have mood swings. From what I read on Google, that is normal. Frankly, when studying puberty, I am always curious whether the symptoms are normal or not.”* (Diary Juanka, grade 8)

Online information regularly supplemented existing information that lacked detail or applicability. For example, as Zoe shared in relation to her menarche experience:

*“The first time I got my period, I did not know what menstruation was. I asked my Ibu [mother] about it. Because I was not satisfied with the answer, I took my phone to find out on the internet what menstruation is. Because I was still curious about it, I tried to find other answers, and I looked for what I could or could not do during menstruation.”* (Diary Zoe, grade 8)

Equally, supplementation occurred when other information was not provided when needed or if information providers, such as mothers, were unavailable.

Online learning provided participants with an increased vocabulary to make sense of and talk about their menstrual symptoms and experiences, such as discharge or changes in blood colour. Searches and encounters opened new ways of understanding symptoms and provided new words to search further, to continue discussions in other spaces, or to make sense of future information. However, participants required basic menstrual knowledge and the ability to label or describe symptoms to initiate a suitable and directed search and minimise unwieldy search results. An absence of a pre-existing vocabulary to search, or masking a link between the symptoms and menstruation, could amplify the existing risk of finding stressful and overwhelming information online. As one participant shared with the group:

*“Like I googled “why do I have pain in my stomach” without stating “menstruation”. Then, Google said that is a sign I will die soon.”* (GD 5, site A)

In some cases, online information was seen to cause worry and complicate otherwise normal experiences. The groups expressed stress at the possibility that their symptoms were signs of disease that required medical attention. For example, in an interview, Julie described her experience looking for personalised information on menstrual discomfort and flow; she states, “*at that time, I was looking for why when menstruating there will always be a stomachache [cramps] and why my menstruation was not heavy. Then, the answer from google was cyst, cancer, and tumour”* (Interview, Grade 7).

Stress and worry could be more pronounced when participants did not – due to shame, lack of interest, or lack of vocabulary – seek further validation of online information or support through other sources such as parents, siblings, friends, teachers, or healthcare workers. Some participants acknowledged the risk of over diagnosing or “overthinking” (GD 4, site B) because of Google and understood this as a deterrent to seeking health information online.

This was also the case in TikTok, where participants suggested that some medically focused content sparked worry and contained excessive or inappropriate information. Encountering such information without support, a previous experience to make sense of, or a baseline understanding to build upon could lead to worry and overthinking rather than informing their current or future experiences.

**HS:** *After watching [the video on TikTok], for example, when you next have your menstruation, will you recognise if there are any clot*s?

**Participant:** *paranoid.*

**KPY:** *paranoid? Why?*

**Participant:** *yeah, because he [TikTok creator] stated that may indicate cancer or melanoid, etc. So I am afraid.* (GD 5, site A)

#### Learning menstrual care practices

Learning and confirming menstrual practices through online information provided practical solutions and information to support menstrual practices, such as pain reduction techniques or self-care. However, not all information was suitable or applicable to the needs of the participants.

On TikTok, creators would share advice based on existing evidence (or misinformation) or personal experiences. Numerous videos demonstrate or act out “how to”, providing fun and relatable instructions otherwise known as “life hacks”.^45^ For example, as a participant stated, “*I always scroll TikTok and look for how to put the pad correctly so there will be no leak”.* (GD 3, site B)

Participants actively searched for additional or alternative solutions when information and support offered by others did not meet their needs. One participant shared how Google can support pain reduction techniques and supplement other practical information, particularly from mothers: “*My mum usually says, ‘Just go to sleep’ but when I try to sleep, it is just pain, so I look for another solution from Google”* (GD 1, site B). Or, as Zoe described in her diary, she builds upon the support from her mother to develop more detailed strategies for self-care:

*“Drinking a lot of water … if something happens, I tell my mum. I got the information on how to take care of myself from the internet. I also look for it on Google, about how to take care of myself and reproductive organs and what I can do during menstruation and don’ts.”*

(Diary Zoe, Grade 8)

In other situations, participants would first use Google to find quick and timely solutions and then follow up on this information with further support from mothers.

**Participant:** *okay this is an example, when we are in school and we have cramps, we don’t have any choice, we cry because it hurts so much.*

**KPY:** *then, you find it on Google?*

**Participant**: *yes, that is correct. And then in the break time, we can phone our mum.*

(GD 1, site B)

Or, as Cathlyn suggested in her interview, the information online was a good start in providing details on suitable pad sizes for her menstrual flow. However, this information was not personalised enough to account for her preferences and needs, so further support was sought from her mother to refine her menstrual practices:

*“There are the centimetres (size) on the pads and also, and I searched it on Google. Is that long or not? So maybe because of Google I used the 32 cm. Then, at first, I used the one with no wings, but I felt uncomfortable, so yeah, my mum told me to change it to the one with wings.”* (Interview Cathlyn, grade 9)

Despite these possibilities, online information was sometimes unsuitable or impractical. The vast array of solutions was, at times, overwhelming and did not always fit the needs or preferences of participants. However, participants discussed how they decided what to adopt and test and what to ignore. As one participant told us when we asked if she tried the pain reduction technique, she found, “*no because I think it doesn’t make sense. Like it is stated that I need to drink ginger water. And I think it is weird”* (GD 3, site B).

## Discussion

This study explored how adolescent schoolgirls in Bali, Indonesia, access and encounter menstrual health information online and how they use this information to meet their needs. We found that participants connected with online information through two pathways: active information seeking – tracing websites through search engines such as Google, and incidental encounters, predominantly through the social media platform of TikTok. Participants used this information in various ways, resulting in opportunities and risks for menstrual health learning. Online information can potentially support adolescents’ rights, such as the right to health or information; however, we found it also poses risks and harms that may jeopardise these rights. We found that online information helped to debunk myths and restrictions, make sense of participants’ new or ongoing menstrual experiences, and enable them to learn, refine, and test menstrual care practices. Equally, online information could perpetuate misinformation, be a source of stress and worry, or offer unsuitable strategies for menstrual care.

### Online learning is relevant to menstrual health

Our research shows that informal online learning, through web-based searches or social media, is an important menstrual information source for adolescent girls in Bali, Indonesia. Our findings align with similar studies, mostly among populations in the Global North, that demonstrate the role of informal online sources for broader health information, particularly among young people.^4,24,26^ Similar to Lupton,^2^ we found that even with the emergence of specific digital health technologies – for example, menstrual tracking apps ­­– there was regular use of “older technologies” like web searching through engines such as Google. This was alongside the increasing influence of social media video platforms, such as TikTok. In addition, we found striking similarities between the actions participants used to seek and encounter menstrual health information and the actions used by young people in contexts such as Australia for broader online health information.^4,26^ These cross-contextual similarities emphasise the growing global relevance and utilisation of online health information among young people in various contexts. However, while mechanisms and platforms like TikTok are worldwide, creating borderless flows of information and global trends, the content accessed and motivations for use are equally locally grounded and specific to the context of the users and their health needs. The content participants encountered was tailored to the Indonesian audience and delivered in Bahasa Indonesian with reference to specific practices and challenges that resonated with the participants. This underscores the need to pay attention to the diversity of online health information-seeking actions among young people globally, particularly in low- and middle-income settings where mobile connectivity is rapidly increasing, simultaneously presenting opportunities and risks.

### Informal online learning is self-directed and perceived as accessible and private

We also found that informal online learning for menstrual health among our study population was “self-directed”.^5^ Based on their needs and interests, participants sought personalised, relatable, immediate, and private information. Online information may support adolescents’ right to seek and receive information through the media of their choice.^46^ This corresponds with findings from studies among young people concerning general or sexual and reproductive health online information-seeking motivations and practices.^24,47,48^

Participants shared that online information was favoured to respond to specific menstrual health questions or symptoms. This was particularly when compared to information offered irregularly through schools or by mothers at menarche, which could be challenging to make sense of or apply later when needed. Furthermore, the format of online information, especially the short videos encountered in TikTok, was convenient, engaging, and relevant, capturing participants’ attention and encouraging recall. Taba et al.^4^ also found that young people in Australia perceived incidental health information through social media as helpful, as it could provide “useful” and “more accessible” opportunities to learn about health.

### Online learning for menstrual health is specific and faces unique challenges

The content, motivations and use of online information mirrored participants’ physical, emotional, and social menstrual experiences and were specific to the gaps and challenges of menstrual health education in Indonesia. The social media information participants recalled and the information they searched or googled reflected their menstrual health needs and questions: a desire for general information on the menstrual cycle, practical information to support their day-to-day menstrual experiences such as pain and discomfort, and information that can verify existing information, specifically related to myths and misinformation. These themes are consistent with common offline menstrual health questions.^49^ Participants’ menstrual health learning practices and motivations were rarely exclusively focused on a singular health concern. Rather, participants would draw on online information to support an immediate (but frequently repeated) concern, like pain, whilst also seeking or encountering information that enabled them to make sense of their ongoing and cyclical menstrual experiences.

Pervasive misinformation, unsubstantiated rules and restrictions, and myths present a specific menstrual health challenge for many young people. Our discussions found that, in some cases, online information helped to debunk misinformation. Participants also used “evidence” to question rules and restrictions, particularly from parents. Like Southerton and Clark,^50^ we found that TikTok offered a space and specific functions for creators (and users) to critically engage with circulating health misinformation and debunk everyday restrictions. However, we found that in some cases, it also amplified and generated novel ways of communicating existing menstrual taboos through ghost story-related content.

### Online information is not a standalone solution

Our findings show that online information interacts with existing information and continuously engages with other (offline) sources. Participants often sought online information when other information was insufficient or inaccessible. Across our study sites, we noted a particular gap in school-based education to offer relatable and applicable content on menstrual health.

In some cases, we found that participants relayed the information they found online to other sources, such as friends or mothers, seeking further support, validation, negation, or simply continuing the conversation due to interest. Other studies with populations in the Global North have found young people often follow up on online information with parents or caregivers.^2,4^ For our study, and specifically for menstrual health, we found the action of following up supported participants and provided opportunities to discuss and share menstrual experiences, receive interpersonal support, further “test” information or refine self-care strategies. This highlights the crucial role parents or adult caregivers play in supporting adolescents in exercising their right to information and health, alongside the obligation to protect adolescents from harm or risks prevalent in digital spaces.

### Online information presents both opportunities and risks

Online learning was a source of care, support, connection, and personalised information, as well as a source of misinformation and worry. Participants acknowledged the vast amount of online information and the possibility that results would be inappropriate, incorrect, or lead to “overthinking”. Freeman et al.^34^ also identified this as a barrier to online information across numerous studies in their systematic review, resulting in participants “expressing frustration” and feeling “overwhelmed”. However, our findings also show that online spaces have the potential to expand menstrual health learning opportunities and foster dialogue. Much of the menstrual health content shared by participants resembled edutainment: fun and engaging content with an educational tone or goal.^50^ Equally, visuals of menstrual processes, such as blood clots or discharge or acting out the severity of menstrual pain, could help break down the perception that experiences are abnormal, “disgusting”, or should not be discussed. Others have also identified and argued for the potential role of online spaces and social media learning, sharing and activism for sexual and menstrual health.^21,51^ We propose that these possibilities hold particular significance for menstrual health learning among young people in settings such as Indonesia, where mobile connectivity is rapidly increasing, and comprehensive menstrual health education through school-based programmes, families, and communities remains limited.

### Limitations

This study captured the experiences of a small group of adolescent girls in Bali. Participants were predominantly from urban areas and Balinese Hindu backgrounds. While we did not directly collect data on participants’ religion, we understood that most participants were from Balinese Hindu backgrounds, which informed many of their menstrual experiences. Except for one rural site, most participants attended schools in urban areas. We imagine girls in other regions of Indonesia and Bali, particularly in rural or remote areas, may have different experiences. Furthermore, all participants had access to personal internet-connected mobile devices. Additional studies are needed to understand the role of online information for menstrual health in other communities, particularly for young people in areas that are assumed to be less connected, as well as the influence of online menstrual health learning among other populations, such as adult women or mothers. Lastly, we required all participants to seek parental consent; this may have excluded participants who did not feel comfortable discussing menstruation with their parents or adolescents whose parents denied consent. Future research could carefully consider how to promote broader inclusion while ensuring the safety and comfort of participants, schools and families.

The study was participatory, ethnographic and part of a broader PhD research project; therefore, it was not within its scope to measure knowledge levels or assess the credibility and reliability of the content participants encountered. Future studies could review online content for menstrual health to assess the reach, quality of the information, and prevalence of misinformation.

## Recommendations and conclusion

Research and policy on menstrual health learning focuses on school-based education. While school-based education covering – at a minimum – the biological and practical domains of menstrual health is vital, it only constitutes one of many menstrual health learning sources. Adequate information is required to achieve menstrual health and support adolescents in realising their fundamental human rights, including the right to health and education.^6^ Our research has shown that participants had a number of questions about menstrual health, indicating the need for further information to address both basic biological facts and unique personal experiences. However, singular sessions are unlikely to sufficiently respond to a variety of young people's questions about their bodies or provide the information needed to support their menstrual health over time.

We echo other studies to emphasise the need to prioritise digital media education and critical health literacy.^4,51,52^ Many young people in Indonesia have high-level digital capacities, exceeding the skills of many adults, such as teachers and caregivers. Equally, as we have demonstrated, menstrual health misinformation does not solely begin or remain in online spaces. Therefore, digital media education and critical health literacy go beyond ensuring young people adequately use digital and online tools. Rather, efforts should recognise the existing skills of young people and encourage critical and constructive engagement with health information and content. Integrating this into menstrual health education should involve collaborating with digital engagement and online safety practitioners to learn from best practices. Ongoing programmes could also provide structured opportunities to discuss and unpack the content or information young people seek and encounter online. Like Goodyear and Armour^24^, our research highlighted the need for adults and mentors to engage with and develop an understanding of young people’s digital health practices. Programmes or research could test the results of assigning accessible and reliable “follow-up” sources, such as a teacher or school staff member, parent, peer, or healthcare worker. These sources could offer pathways to fact-check and follow-up online information, connect young people to support, and encourage open communication about digital content and menstrual health needs.

Enhancing menstrual health among young people requires a commitment to understanding their diverse lived experiences and evolving needs. It also requires that policy, programme and decision-makers recognise the role of online information and meet their obligation to foster environments where young people can safely exercise their right to seek and access diverse and quality information from a range of sources.^46^ Creators, governments and health and education providers should encourage the availability of such content while proactively monitoring and addressing harmful or inaccurate content. Developments such as the Oky App (UNICEF), a period tracker with educational features, represent growing recognition of the need to expand how menstrual health information is delivered in Indonesia and other contexts. While progress is under way, there is space for appropriate online sources and content that young people can easily access or encounter in their language. This could be supported through further cross-sectoral collaboration between practitioners, experts, content creators and developers.

While informal online information presents unique risks, it is a relevant source of learning and provides young people with immediate menstrual health information. In contexts such as Indonesia, where persistent barriers limit comprehensive sexual and reproductive health education and hinder adolescents’ realisation of their rights to health and information, online learning may serve to address gaps and support ongoing learning. Moving forward, the task is to understand how to collaborate for critical and positive engagement with online information while minimising possible risks.
